# AMPA receptors in the evolving synapse: structure, function, and disease implications

**DOI:** 10.3389/fnsyn.2025.1661342

**Published:** 2025-10-10

**Authors:** Fleming Francis, Dewan Chettri, Deepak Nair

**Affiliations:** Centre for Neuroscience, Indian Institute of Science, Bengaluru, India

**Keywords:** iGluRs, AMPA receptor (AMPAR), synaptic plasticity, TARP, neurodegenerative diseases, neurodegeneration

## Abstract

Synapses, once considered static conduits for neuronal signals, are now recognized as dynamic, multifunctional structures critical to brain function, plasticity, and disease. This evolving understanding has highlighted the tripartite nature of synapses, including pre-synaptic terminals, post-synaptic compartments, and regulatory glial elements. Among excitatory synapses, glutamatergic transmission dominates, with AMPA receptors (AMPARs) playing a central role in fast synaptic signaling. AMPARs are tetrameric, ligand-gated ion channels that mediate rapid depolarization and are tightly regulated by subunit composition, trafficking, and interactions with scaffolding and signaling proteins. Their activity-dependent modulation underpins key processes such as long-term potentiation and depression, central to learning and memory. Importantly, dysfunctions in AMPAR expression, localization, or signaling are increasingly linked to neurological and psychiatric disorders including autism spectrum disorders, epilepsy, schizophrenia, and Alzheimer's disease. This review discusses AMPAR biology in the context of synaptic organization, highlighting recent advances and ongoing challenges in understanding their roles in health and disease.

## Introduction

Over the past three decades, our understanding of synapses, the fundamental units of communication between neurons, has evolved dramatically. Once regarded as static junctions responsible solely for transmitting signals across neurons, synapses are now recognized as dynamic, multifunctional structures, finely tuned in space and time. Advances in molecular and imaging technologies have revealed that synaptic composition and organization are not fixed but instead undergo rapid and localized changes with nanometer-scale spatial precision and submillisecond temporal resolution (Nair et al., 2013; Archibald et al., 1998; Fukata et al., 2024). This transformation in perspective has led to the recognition that synapses are not merely neuronal interfaces; they are also the fundamental building blocks of neuronal communication. They are now understood as tripartite functional units composed of the pre-synaptic terminal, the post-synaptic membrane, and a third regulatory element, often glial cells, particularly astrocytes (Archibald et al., 1998; Szatkowski et al., 1990; Bevan et al., 1973; Dennis and Miledi, 1974; Villegas, 1972). These non-neuronal cells are not passive bystanders; rather, they actively modulate synaptic strength and contribute to the homeostatic regulation of both the immediate synaptic environment and broader neuronal networks (Perea et al., 2009; Kettenmann et al., 2025). This insight has expanded our conceptual framework of how information is processed and stored in the brain and has opened new avenues for exploring mechanisms of synaptic dysfunction in disease.

A significant portion of this new understanding has come from studies of excitatory synapses in the mammalian brain. Among the various neurotransmitter systems that mediate excitation, glutamatergic synapses predominate, accounting for approximately 80–90% of excitatory synaptic activity (Somogyi et al., 1998; Shinohara and Hirase, 2009). These synapses are predominantly localized to the dendritic spines, with small, actin-rich protrusions emerging from the dendritic shaft (Weber et al., 2016; Segal, 2005). Each spine typically receives input from a single pre-synaptic bouton, which contains synaptic vesicles filled with glutamate. Upon stimulation, glutamate is released into the synaptic cleft, where it binds to receptors on the post-synaptic membrane. This event triggers the depolarization of the spine and initiates the post-synaptic signaling cascade that determines the likelihood of generating an action potential (Qu et al., 2009; Ikeda and Bekkers, 2009). The structural and functional compartmentalization provided by dendritic spines enables localized signal processing, allowing neurons to integrate thousands of synaptic inputs with remarkable specificity and plasticity (Tecuatl et al., 2021; Drachman, 2005). Over the last two decades, detailed molecular dissection of synaptic architecture has revealed a complex network of proteins involved in synaptic transmission, plasticity, and stability. Key among these are the molecular sensors and receptors that detect the arrival of neurotransmitters in the synaptic cleft. These include ionotropic receptors such as AMPA and NMDARs, which mediate fast excitatory transmission, as well as metabotropic glutamate receptors that modulate slower, longer-lasting synaptic responses (Watkins and Evans, 1981; Conn and Pin, 1997; Barnes and Henley, 1992). The trafficking, localization, and functional tuning of these receptors are tightly regulated by scaffolding proteins, kinases, and other signaling molecules that collectively form the post-synaptic density (PSD)—a dense, protein-rich structure critical to synaptic integrity. The pre-synaptic machinery is equally intricate, involving vesicle docking, priming, and calcium-dependent fusion processes mediated by proteins such as synaptotagmins, SNAREs, and complexins (Sheng and Kim, 2011; Schoch and Gundelfinger, 2006; Scannevin and Huganir, 2000; Dani et al., 2010; MacGillavry et al., 2013; Fukata et al., 2013). The coordinated activity of these proteins ensures that neurotransmitter release is both rapid and precisely timed. Importantly, many of these molecules are subject to activity-dependent regulation, providing a mechanistic basis for synaptic plasticity phenomena such as long-term potentiation (LTP) and long-term depression (LTD), which underlie learning and memory (Szatkowski et al., 1990; Bevan et al., 1973; Barnes and Henley, 1992).

Glutamate binds with Glutamate receptors and functions as an excitatory neurotransmitter in the Central Nervous System. Classically, Glutamate receptors are classified into two major types: ionotropic glutamate receptors (iGluRs) and metabotropic glutamate receptors (mGluRs). iGluRs, including α-Amino-3-hydroxy-5-methyl-4-isoxazolepropionic acid (AMPA), N-methyl-D-aspartate (NMDA), and kainate receptors, are ligand-gated ion channels that directly affect neuronal excitability (Traynelis et al., 2010). On the other hand, mGluRs are G protein-coupled receptors that indirectly regulate neuronal activity through intracellular signaling pathways (Niswender and Conn, 2010). While early studies in synaptic biology emphasized how these molecular components ensure reliable neuronal signal transmission, contemporary research increasingly highlights their roles in neurological and psychiatric disorders. Our review focuses more on the roles of glutamate receptors, particularly AMPA (α-amino-3-hydroxy-5-methyl-4-isoxazolepropionic acid) receptors, which are key mediators of fast excitatory synaptic transmission in the central nervous system. Discovered as ligand-gated ion channels responsive to the neurotransmitter glutamate, AMPARs are tetrameric complexes typically composed of GluA1-4, with distinct functional properties governed by their subunit composition, post-translational modifications, and interactions with auxiliary proteins (Miguez-Cabello et al., 2025; Greger et al., 2017; Certain et al., 2023). Functionally, AMPARs are critical for synaptic plasticity mechanisms such as LTP and LTD, which underlie learning and memory (de Leon-Lopez et al., 2025; Citri and Malenka, 2008). Their rapid trafficking to and from the synaptic membrane enables dynamic regulation of synaptic strength in response to activity patterns, positioning them as central molecular integrators of experience-dependent neural adaptation. Importantly, dysfunctions in AMPAR expression, localization, or signaling have been implicated in a wide range of brain disorders. Mutations in AMPAR subunits or their associated scaffolding proteins have been linked to autism spectrum disorders, intellectual disability, epilepsy, and schizophrenia (Yonezawa et al., 2022; Jimenez-Sanchez et al., 2024; Hanada, 2020). Moreover, in neurodegenerative diseases like Alzheimer's, altered AMPAR trafficking and synaptic localization contribute to early synaptic failure and cognitive decline, often preceding neuronal loss (Zhang et al., 2022; Babaei, 2021). As research continues to uncover the complex modulation of AMPARs across developmental, physiological, and pathological states, they remain a focal point in efforts to elucidate and correct the molecular basis of cognitive and behavioral dysfunctions. In this review, we will track a brief recap of the AMPA subtype of glutamatergic receptors, their implication in health and diseases, and current challenges and advances that allow us to dissect the roles of these molecules from the perspectives of synapses and synaptic organization.

## Glutamate in the brain and its function

L-glu is the major excitatory neurotransmitter involved in functions such as learning and memory, long-term potentiation, and synaptic plasticity (Zhou and Danbolt, 2014; Tapiero et al., 2002; Petroff, 2002; McKenna, 2007; Fairman and Amara, 1999; Erecinska and Silver, 1990). A kilogram of brain tissue contains 5–15 mmol L-glu, depending on the region, which far exceeds concentrations of all other amino acids (Schousboe, 1981). Neurons also show very high activity when it comes to L-glu uptake (Stern et al., 1949). L-glu's excitatory effect was later discovered as well (Fonnum, 1984; Curtis et al., 1960, 1959). L-glu release from pre-synaptic vesicles occurs via Ca^2^^+^-dependent exocytosis triggered by voltage-gated calcium channels (Meldrum, 2000; Anderson and Swanson, 2000). With vesicular L-glu concentrations reaching ~100 mM, the release of a single vesicle generates an excitatory post-synaptic potential (EPSP) (Meldrum, 2000). Virtually all nervous system cells express at least one glutamate receptor subtype (Vernadakis, 1996; Shelton and McCarthy, 1999; Bergles et al., 2000). Studies involving Glutamate receptor-mediated mechanisms have not only led to the development of treatments for Glutamate-related neurodegenerative diseases such as Alzheimer's, Parkinson's disease, and multiple sclerosis, but also a basic understanding of synapses and how the brain controls structure-function relationship from molecular, and cellular levels that have repercussions in cognitive processes (Pizzi et al., 2005).

## Glutamate receptors

Glutamate receptors comprise two structurally distinct families of transmembrane proteins: iGluR and mGluRs (Watkins and Evans, 1981; Barnes and Henley, 1992). IGluR facilitate rapid synaptic signaling. Structurally, iGluRs function as tetrameric ligand-gated ion channels with distinct pharmacological profiles and electrophysiological characteristics (Traynelis et al., 2010). Excessive or prolonged activation of iGluRs and other post-synaptic signaling components can induce excitotoxicity, a process strongly linked to neurodegenerative diseases and nervous system injuries (Danbolt, 2001; Cortese and Phan, 2005; Bains and Hall, 2012). mGluRs, in contrast, are G-protein-coupled receptors (GPCRs) that activate downstream signaling cascades or modulate cation influx upon L-glu binding, regulating synaptic efficacy and plasticity (Conn and Pin, 1997; Tikhonov and Magazanik, 2009). These receptors are widely distributed in the central and peripheral nervous systems and have been implicated in maintaining homeostasis across multiple organ systems (Niswender and Conn, 2010; Julio-Pieper et al., 2011).

**Figure 1:**
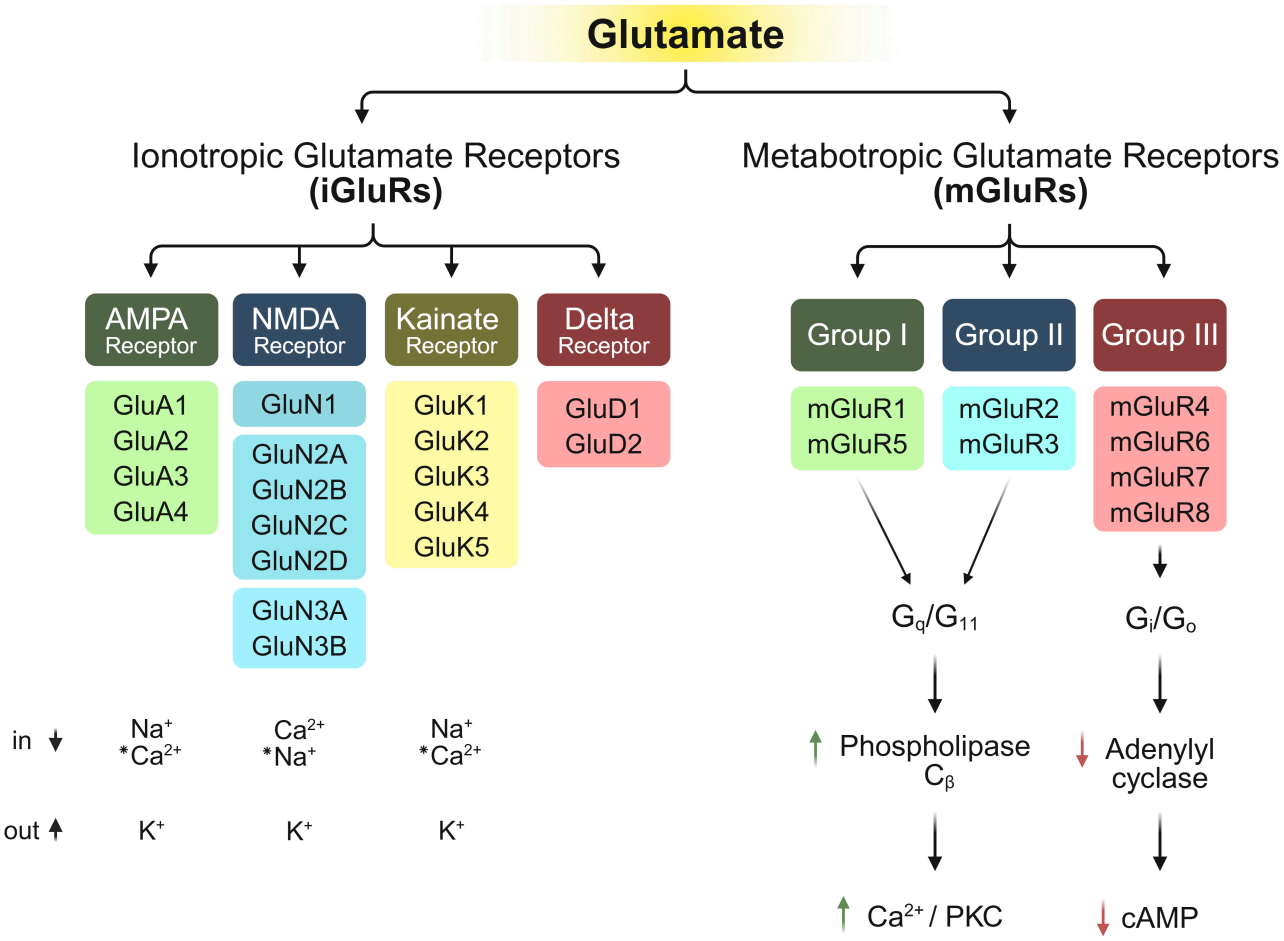
Structural and functional diversity of iGluR subtypes and their respective permeability to ions (Watkins and Evans, 1981; Barnes and Henley, 1992; Traynelis et al., 2010; Lomeli et al., 1993), along with the downstream signaling of mGluRs upon binding of mGluRs (Conn and Pin, 1997; Coutinho and Knopfel, 2002). “*” indicates special conditions for the conduction of these ions.

Extensive pharmacological research conducted between 1980 and 2000 established the canonical classification system for iGluRs, dividing them into three distinct functional categories: (1) AMPA-sensitive receptors, activated by α-amino-3-hydroxy-5-methyl-isoxazole-propionic acid (GluA1-GluA4), (2) kainate-sensitive receptors, activated by kainic acid (GluK1-GluK5), (3) NMDA-sensitive receptors, activated by N-methyl-D-aspartate (GluN1, GluN2A-D, GluN3A-B), and the orphan glutamate receptors (4) (GluD1 and GluD2) that formed a separate phylogenetic cluster classified as Delta receptors due to their unique sequence features and lack of known agonists, bringing the total to four major groups (Watkins and Evans, 1981; Yamazaki et al., 1992; Watkins et al., 1990; Stevens, 1986; Simon et al., 1976; Nowak et al., 1984; Lomeli et al., 1993; Honore et al., 1982; Henley et al., 1989) (Figure 1).

As mentioned above, excitatory post-synaptic currents were initially categorized according to their kinetic and pharmacological properties, distinguishing between currents mediated by AMPA-, kainate-, and NMDA-type glutamate receptors (Watkins and Evans, 1981). However, there was a major drawback to this method of classifying glutamate receptors. There are subunits classified under glutamate receptors that do not bind glutamate. Subunits GluN3A and GluN3B of the NMDA family do not bind L-glu, and NMDA receptors consisting of them are insensitive to glutamate and instead bind glycine to form excitatory glycinergic receptors (Yao et al., 2008; Ulbrich and Isacoff, 2008; Chatterton et al., 2002; Al-Hallaq et al., 2002). Also, five of the 18 vertebrate iGluR subunits, GluN1, GluN3A, GluN3B, GluD1, and GluD2, also bind glycine other than glutamate, while also endogenously interacting with the D-serine in multiple brain regions (Naur et al., 2007; Kumar et al., 2023; Hansen et al., 2021; Alberstein et al., 2015). Hence, now the classification has shifted to a more sequence homology-based approach.

The classes have different electrophysiological characteristics as well. AMPARs display rapid desensitization kinetics, while kainate receptors undergo more prolonged desensitization (Mosbacher et al., 1994; Erreger et al., 2004; Castillo et al., 1997). NMDARs possess unique activation requirements, demanding concurrent conditions: (1) glycine/serine binding, (2) pre-synaptic L-glu release, and (3) post-synaptic membrane depolarisation, removing a magnesium block from the channel pore, allowing the exchange of ions. This triple-gating mechanism establishes NMDARs as molecular coincidence detectors, particularly crucial for initiating molecular reorganization important for persistent long-term changes like LTP. They also (Tang et al., 1999; Kleckner and Dingledine, 1988; Johnson and Ascher, 1987). Recombinant delta receptors don't directly open on binding of agonists but have been shown to conduct depolarising current when induced by mGluRs (Wo and Oswald, 1995).

## Ionotropic glutamate receptors: structure, function, and assembly

**Figure 2:**
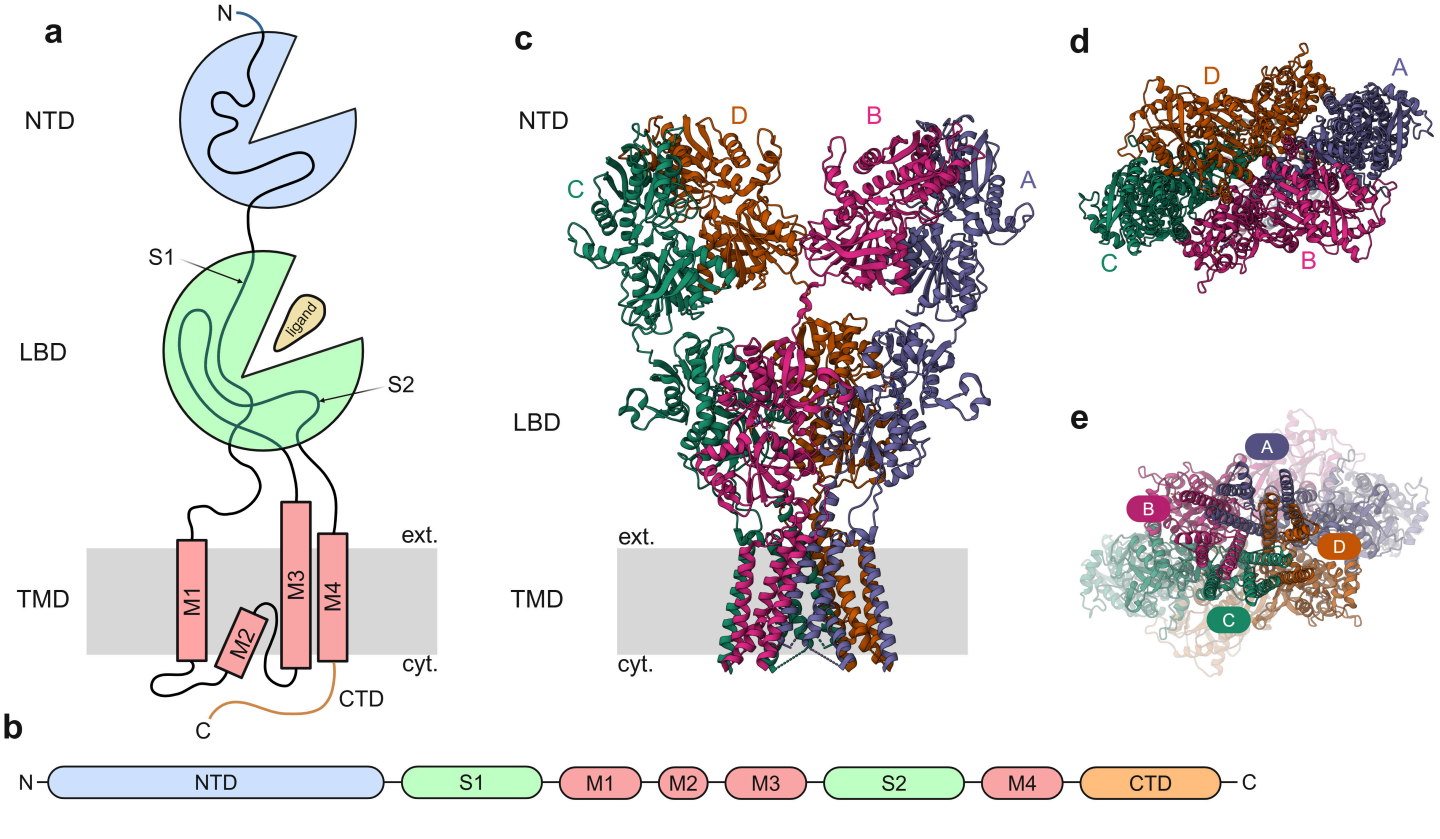
General architecture of iGluRs. **(a)** Cartoon depicting general iGluR architecture with the domains NTD, LBD, and TMD. **(b)** Subunit polypeptide chain linear representation. **(c)** 3D structure of GluA2 homomer [PDB ID: 3KG2, (Sobolevsky et al., 2009)] with the different subunits marked. **(d)** The NTD, viewed from the outside of the cell toward the cytosol. **(e)** The TMD, viewed from the cytosol toward the exterior of the cell, with the channel pore visible.

The molecular architecture of iGluR subunits consists of four distinct structural domains: (1) an extracellular N-terminal/amino-terminal domain (NTD/ATD), (2) an extracellular ligand-binding/agonist-binding domain (LBD/ABD), (3) a transmembrane domain (TMD), and (4) an intracellular carboxyl-terminal domain (CTD) (Sobolevsky et al., 2009; Laube et al., 1998) (Figure 2). The LBD forms through the interaction of two discontinuous polypeptide segments (S1 and S2) that flank the membrane-spanning regions. The TMD contains three transmembrane helices (M1, M3, M4) and a membrane-reentering loop (M2), while the CTD mediates subcellular targeting and regulatory post-translational modifications (Warnet et al., 2021; Evans et al., 2019; Anggono and Huganir, 2012). Ligand binding induces a conformational change in the LBD that mechanically couples to the TMD, causing structural rearrangements that open the ion conduction pathway. This gating mechanism, along with the presence of key amino acids that make up the selectivity filter of the channel pore, permits selective permeation of Na^+^, K^+^, and Ca^2+^ ions across the membrane (Twomey and Sobolevsky, 2018).

iGluRs function as tetrameric assemblies (Sobolevsky et al., 2009; Laube et al., 1998; Rosenmund et al., 1998; Nakagawa et al., 2005; Mano and Teichberg, 1998) with strict subunit selectivity, ensuring receptors form exclusively from members of the same pharmacological class (Monyer et al., 1992; Leuschner and Hoch, 1999; Kuusinen et al., 1999; Ayalon and Stern-Bach, 2001; Ayalon et al., 2005). This assembly principle maintains the functional specificity of AMPA, kainate, NMDA, and Delta receptor subtypes.AMPARs have flexible assembly properties, with GluA1-GluA4 subunits capable of forming both homomeric as well as heteromeric complexes (Hollmann and Heinemann, 1994; Herguedas et al., 2013; Bowie, 2012). AMPARs comprise both calcium-impermeable (CI-AMPARs) and calcium-permeable (CP-AMPARs) subtypes, with CP-AMPARs distinguished by their lack of GluA2 subunits. GluA2-lacking AMPARs are permeable to calcium, whereas those containing GluA2 subunits are calcium-impermeable (Burnashev et al., 1992). But recently it has been shown that GluA2-containing AMPARs are also permeable to calcium (Miguez-Cabello et al., 2025). CP-AMPARs exhibit unique biophysical and pharmacological signatures, enabling their functional identification. Studies have demonstrated their critical involvement in multiple forms of synaptic plasticity, like LTP (Park et al., 2019), LTD (Sanderson et al., 2016), and homeostatic plasticity (Sutton et al., 2006; Sanderson et al., 2018). In contrast, NMDARs exhibit strict subunit requirements: functional channels necessitate two obligatory GluN1 subunits combined with either two GluN2 subunits or a combination of GluN2 and GluN3 subunits (Ulbrich and Isacoff, 2008, 2007; Schorge and Colquhoun, 2003). Kainate receptors display intermediate assembly characteristics: GluK1-GluK3 subunits can form homomers or heteromers among themselves, while GluK4 and GluK5 require co-assembly with at least one GluK1-3 subunit for functionality (Reiner et al., 2012; Meyerson et al., 2016; Jaskolski et al., 2005; Herb et al., 1992; Cui and Mayer, 1999). The orphan Delta receptors GluD1 and GluD2 subunits assemble into homotetrameric complexes that function as trans-synaptic adhesion molecules and ion channels (Naur et al., 2007; Mayat et al., 1995; Kohda et al., 2000).

## Metabotropic glutamate receptors

Eight members of the G-protein-coupled mGluR family have been identified to date, namely mGluR1–8. Based on pharmacological properties, second messenger coupling, and sequence homology, mGluRs are classified into three groups: Group I (mGluR1 and mGluR5), Group II (mGluR2 and mGluR3), and Group III (mGluR4, mGluR6, mGluR7, and mGluR8) (Yin et al., 2014; Anwyl, 1999) (Figure 1). Group I mGluRs primarily signal through G_q_/G_11_ proteins, activating phospholipase C_β_, whereas Group II and Group III mGluRs couple to G_i_/G_o_ proteins (Conn and Pin, 1997; Coutinho and Knopfel, 2002; Anwyl, 1999; Valenti et al., 2002; Hermans and Challiss, 2001).

Group I mGluRs typically couple to G_q_/G_11_ to activate phospholipase C_β_, triggering phosphoinositide hydrolysis that results in the formation of inositol 1,4,5-trisphosphate (IP_3_) and diacylglycerol (DAG). This canonical pathway causes the release of intracellular calcium from the ER and activates protein kinase C (PKC). An increase in intracellular calcium also induces inhibitory post-synaptic potentials (IPSPs) via Ca^2^^+^-activated K? conductance (Fiorillo and Williams, 1998). However, these receptors are now known to regulate additional signaling cascades beyond G_q_, including pathways mediated by G_i/o_, G_s_, and G protein-independent mechanisms (Hermans and Challiss, 2001). Their downstream effects vary by cell type, modulating effectors like phospholipase D, multiple protein kinases like casein kinase 1, cyclin-dependent kinase 5, Jun kinase, and key pathways related to plasticity, particularly the MAPK/ERK and MTOR/p70 S6 kinase cascades (Page et al., 2006; Li et al., 2007; Hou and Klann, 2004). Group II and III mGluRs instead primarily signal through G_i/o_ proteins, which typically inhibit adenylyl cyclase while directly modulating ion channels and other effectors via the release of the G protein subunits G_βγ_. Recently, emerging evidence has shown that these receptors also engage in other signaling pathways, including MAPK and PI3 kinase activation (Iacovelli et al., 2002). Even though mGluRs are capable of facilitating synaptic communication by generating prolonged excitatory post-synaptic depolarisations, their principal function lies in fine-tuning neural activity, where they adjust cellular responsiveness, information transfer across synapses, and adaptive changes in synaptic strength.

**Figure 3:**
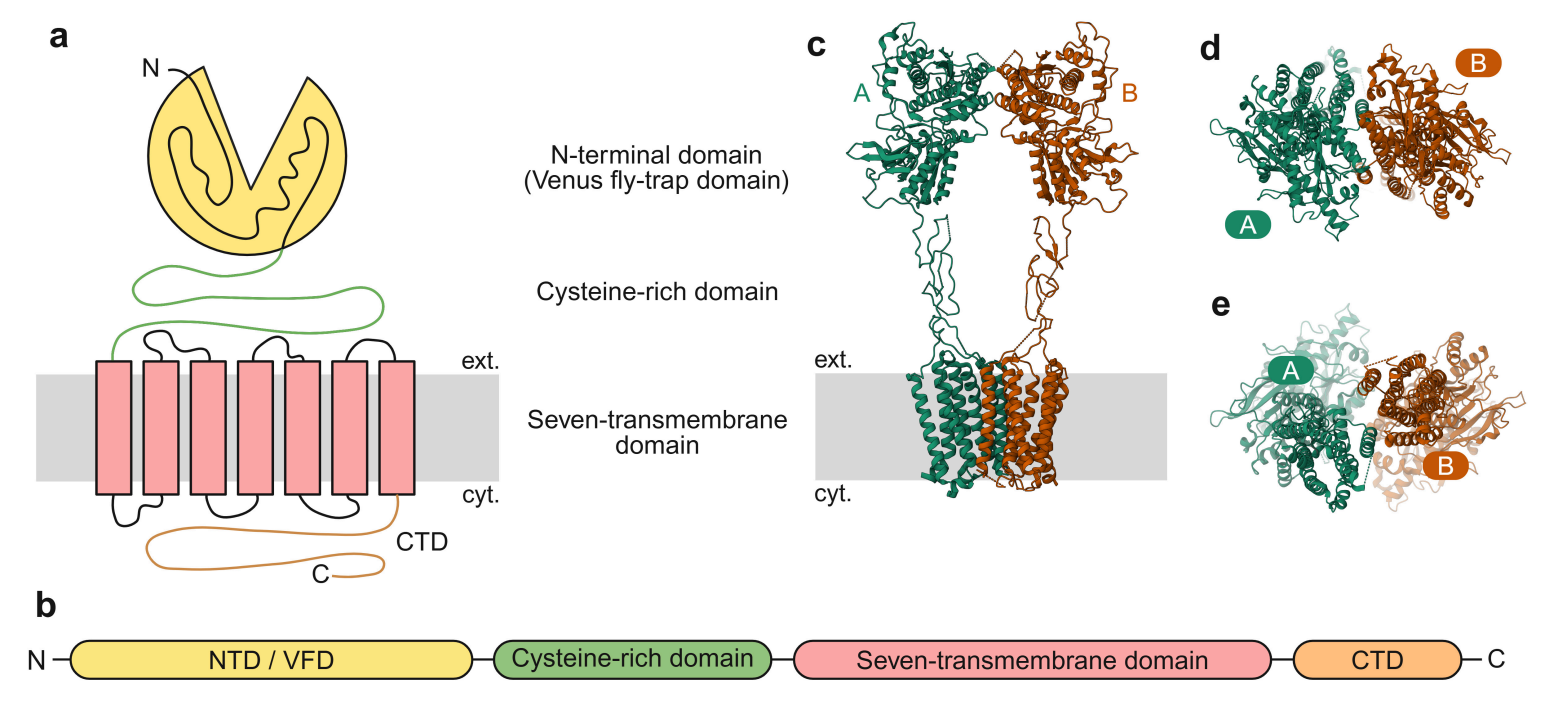
General architecture of mGluRs. **(a)** Cartoon depicting general mGluR architecture with the VFD, CRD, and the seven-transmembrane domain. **(b)** Subunit polypeptide chain linear representation. **(c)** 3D structure of mGluR2 homomer (PDB ID:7MTQ, Seven et al., 2021) with the different subunits marked. **(d)** The NTD/VMD, viewed from the outside of the cell toward the cytosol. **(e)** The seven-transmembrane domain, viewed from the cytosol toward the exterior of the cell.

Structurally, mGluRs are dimeric and possess an extensive extracellular N-terminal region known as the Venus flytrap domain (VFD). This structural element harbors the L-glu binding pocket and plays an essential role in both homomer and heterodimer formation among these receptors (Pin et al., 2003; Muto et al., 2007; Kunishima et al., 2000; Jingami et al., 2003) (Figure 3). Ligand binding induces conformational changes that propagate from the Venus flytrap domain (VFD) through cysteine-rich domains (CRDs) to the seven-transmembrane domain and the C-terminal tail (Muto et al., 2007; Rondard et al., 2006). The C-terminal domains of mGluRs play crucial roles in regulating G protein coupling. These regions also undergo alternative splicing, phosphorylation-dependent regulation, and modulatory protein-protein interactions in several mGluR subtypes (Niswender and Conn, 2010). MGluRs exhibit broad expression across the central nervous system, present in numerous major brain areas. They show precise localization patterns, occupying both synaptic and extrasynaptic compartments within neuronal and glial cells. They have also been associated with multiple neuropsychiatric disorders, including Fragile X syndrome, schizophrenia, and autism spectrum disorder (Bhattacharyya, 2016).

## Evolution of glutamate as a major excitatory neurotransmitter

The seminal work of Curtis, Phillis, and Watkins demonstrated L-glu's capacity to induce depolarization in central neurons, establishing its potential as the primary excitatory neurotransmitter in the CNS (Moroz and Romanova, 2021). Evolutionarily, L-glu, ATP, and nitric oxide (NO) acquired neurotransmitter functions early in cellular evolution, with these signaling roles emerging concurrently in primitive cells (Moroz, 2009; Bennett et al., 2009). Substantial evidence indicates that L-glu synthesis occurred on primordial Earth (>3.5–4 billion years ago) under reducing atmospheric conditions, driven by diverse energy inputs. L-glu represents the predominant metabolic intermediate across bacterial and mammalian systems, achieving intracellular levels of 96 mM in *E. coli* (Park et al., 2016), 44 mM in yeast, and 64 mM in immortalized baby mouse kidney (iBMK) epithelial cells (Ohwada and Sagisaka, 1987). Its pool size exceeds 20–40% of the total cellular metabolite content. Within bacterial biofilms, metabolic and electrical signaling networks emerge, with L-glu serving as a critical mediator between central and peripheral cell populations (Moran et al., 2015; Liu et al., 2015). L-glu functions as a versatile nutrient source, supplying carbon, nitrogen, and energy, and consequently, nearly all prokaryotic organisms require L-glu detection to maintain ecological competitiveness across diverse environments.

## Structural evolution of iGluRs

**Figure 4:**
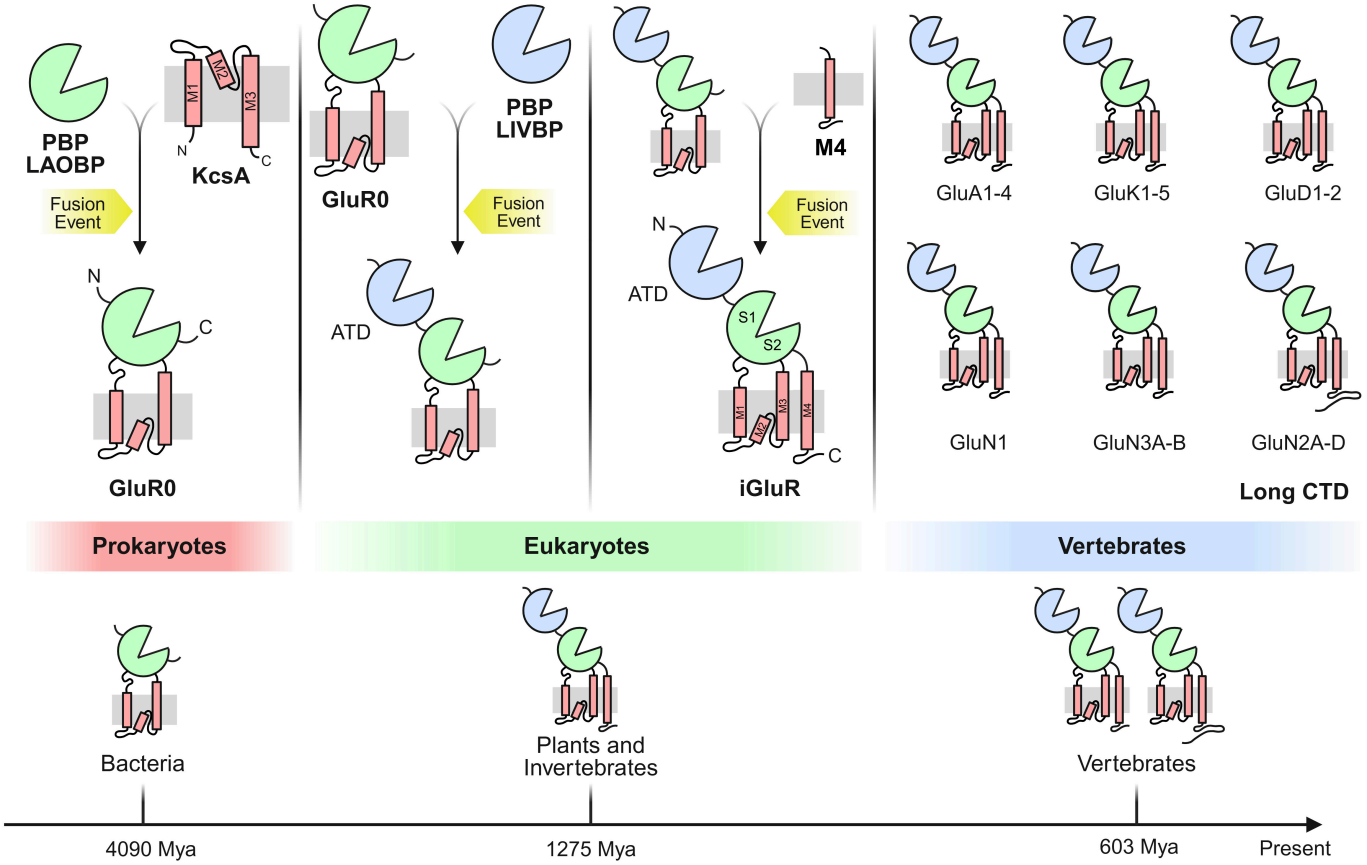
Evolution of the vertebrate iGluR through fusion of multiple prokaryotic genes. A LAOBP (lysine-arginine-ornithine-binding periplasmic protein), forming the ligand-binding domain (LBD), fused with a KcsA potassium channel. Separately, a prokaryotic iGluR (GluR0) fused with a LIVBP (leucine-isoleucine-valine-binding periplasmic protein) and acquired sequences encoding the M4 transmembrane segment and C-terminal domain (CTD), ultimately yielding the domain architecture of vertebrate iGluRs. These fusion events may not have occurred in the depicted sequence. The bottom shows the phylogenetic tree of iGluR-containing organisms, with estimated lineage divergence times annotated below.

Potassium channels likely served as the evolutionary precursors for glutamate receptors, representing a crucial exaptation event. Phylogenetic analyses indicate that potassium channels predate most other ion channel families, evidenced by the remarkable conservation of their transmembrane domains (Yu et al., 2005; Miller, 2000; Arinaminpathy et al., 2003). The co-dependence of potassium and L-glu metabolism supports the proposed dual-origin model for iGluRs suggesting their emergence from (i) ancestral potassium channels and (ii) a nutrient-sensing ligand-binding domain (Tikhonov and Magazanik, 2009; Ger et al., 2010; Felder et al., 1999; De Bortoli et al., 2016). It has been proposed that this complex architecture emerged from the fusion of multiple prokaryotic genes, each adding new domains. The domain organization of iGluRs is conserved across eukaryotes (Alberstein et al., 2015; Scheepers et al., 2016), arising from the fusion of prokaryotic genes (e.g., KcsA + LAOBP). This modular evolution enabled specialization for L-glu signaling, while retaining ancestral roles in nutrient sensing (Chen et al., 1999) (Figure 4).

Eukaryotic iGluRs share an evolutionary lineage with bacterial GluR0-type potassium channels (Arinaminpathy et al., 2003; Schonrock et al., 2019; Kuner et al., 2003). This homology extends to viral K-channel homologs, suggesting lateral gene transfer played a role in iGluR evolution. Prokaryotic iGluR-like structures are found in bacteria, cyanobacteria, and archaea, though these lack the NTD, M4 segment, and CTD present in eukaryotic iGluRs (Tikhonov and Magazanik, 2009; Felder et al., 1999; De Bortoli et al., 2016). The transmembrane domain (TMD) of iGluRs, particularly M1, M2, and M3, shows high structural similarity to the bacterial potassium channel KcsA (K channel of *Streptomyces A*) (Schrempf et al., 1995; Meuser et al., 1999; Chiu et al., 1999). This supports the hypothesis that GluR0 evolved from the insertion of KcsA between the two lobes of a periplasmic binding protein (LAOBP) (Jatzke et al., 2002; Forde and Lea, 2007). Notably, GluR0 remains K-selective, while eukaryotic iGluRs are non-selective cation channels (Kumar et al., 2009; Burnashev et al., 1995). The bilobed LBD (S1/S2) of the Ligand-Binding Domain (LBD) originated from prokaryotic periplasmic substrate-binding proteins (SBPs), particularly the lysine-arginine-ornithine (LAOBP) and leucine-isoleucine-valine (LIVBP) binding families (Jatzke et al., 2002; Forde and Roberts, 2014).

The N-terminal domain (NTD) facilitates receptor tetramerization in the ER (Sia et al., 2007) and helps with interactions with N-cadherins, pentraxins, and ephrins (Saglietti et al., 2007; Paoletti et al., 2000). It also provides binding sites for regulatory ions/small molecules (Masuko et al., 1999; Ehlers et al., 1998). The Transmembrane Segment M4 provides cytoplasmic-facing C-termini, critical for post-translational modifications (Bayés et al., 2014) and scaffolding protein interactions (Salussolia et al., 2011; Husi et al., 2000). It also helps with tetramer stabilization via conserved residues interacting with M1/M3 (Yuan et al., 2005). The M3 domain contains a highly conserved gating motif, maintaining a closed state in eukaryotes (Kohda et al., 2000; Chater and Goda, 2022), unlike bacterial GluR0s, which are passively open.

Major base substitutions also exist between humans and chimpanzees, including GluN3A-D71G (loss of myristoylation) and GluN3B-R727H (gain of phosphorylation), both directly affecting synaptic plasticity, LTP, and LTD (Goto et al., 2009).

## Role in synaptic transmission

Synaptic plasticity is defined by the experience-driven alteration of synaptic transmission efficacy at synapses, which is a fundamental mechanism of neural circuit adaptation and information processing. Synaptic plasticity is of two main types: short-term synaptic plasticity (STP), which lasts for tens to hundreds of milliseconds to several minutes and results in a temporal modification of synaptic efficiency, and LTP, which can have a duration of from minutes to hours, days, or even months. LTD is another form of synaptic plasticity that works antagonistically to LTP, resulting in a progressive weakening of synapses (Citri and Malenka, 2008). The concept of neuronal circuits was first introduced over a century ago by Spanish Nobel laureate Santiago Ramón y Cajal in his book, “Histology of the nervous system of man and vertebrates.” Histology of the nervous system of man and vertebrates (Ramón y Cajal, 1995), Oxford University Press. He identified the axonal growth cone and experimentally disproved the reticular theory (which posited the nervous system as a continuous network), instead demonstrating that neurons were contiguous yet separated (Jones, 1994). This work presented concrete evidence for the “neuron theory,” now widely regarded as the foundation of modern neuroscience.

This foundation was expanded in the late 1940s by Donald Hebb, who theorized that associative memories emerge through synaptic strengthening when pre-synaptic activity coincides with post-synaptic firing (Hebb, 2005). This mechanism of synaptic plasticity, which encodes memories by detecting coincident events, provides a compelling cellular explanation for behavioral learning paradigms like the classical Pavlovian conditioning (Pavlov, 2010). Growing evidence suggests that impaired synaptic plasticity may underlie many neuropsychiatric disorders (Citri and Malenka, 2008). These will be discussed in the latter parts of the review.

The majority of excitatory synapses in the mammalian brain are glutamatergic, where pre-synaptic activity triggers the fusion of glutamate-packed vesicles. The released L-glu diffuses across the ~20 nm synaptic cleft and binds to post-synaptic glutamate receptors (Kerchner and Nicoll, 2008). AMPARs facilitate fast excitatory L-glu signaling in the mammalian CNS by gating rapid sodium channel activity. These receptors dynamically adjust to neuronal activity patterns through swift insertion into or removal from post-synaptic membranes. Synaptic strength strengthens during LTP through increased AMPAR incorporation, while LTD weakens connections via receptor removal (Huganir and Nicoll, 2013).

The discovery of silent synapses fuelled the search for the role of AMPARs in Early LTP. Silent synapses in the CA1 region of the hippocampus are characterized by the absence of detectable excitatory post-synaptic currents (EPSCs) at resting membrane potential, despite receiving pre-synaptic L-glu release. This phenomenon is primarily attributed to synapses containing NMDARs but lacking functional AMPARs under basal conditions. However, these synapses can be functionally “unsilenced” upon post-synaptic depolarisation and induction of NMDA, leading to insertion of AMPARs, which allows previously silent synapses to participate in neuronal communication (Makino and Malinow, 2009; Lledo et al., 1998; Liao et al., 1995; Isaac et al., 1995). These silent synapses play a crucial role in synaptic plasticity and are particularly abundant during early development, decreasing in number with maturation (Lüscher et al., 1999).

AMPAR insertion occurs primarily in perisynaptic zones rather than directly at the synaptic site. From these regions, the receptors are incorporated into the membrane and subsequently diffuse laterally to reach synaptic locations (Penn et al., 2017). This mechanism allows for controlled delivery and integration of AMPARs into the post-synaptic density during synaptic plasticity. The GluA1 subunit is especially crucial for the activity-driven insertion of AMPARs during LTP. AMPARs exhibit high mobility, freely diffusing in and out of synapses until being anchored by PDZ domain-containing scaffolding proteins (primarily PSD-95) at the post-synaptic density. This is followed by synaptic entry via lateral diffusion (Kim et al., 2014).

Late-LTP involves new protein synthesis, including AMPAR subunits and auxiliary proteins, to sustain this now-established synaptic strengthening. Dendritic spines enlarge, and new synapses form, increasing the number of AMPARs (Penzes et al., 2008; Okuno et al., 2012; Murakoshi et al., 2011). In LTD, AMPARs are internalized via endocytosis, weakening synapses (Fiuza et al., 2017; Ehlers et al., 2007; Chowdhury et al., 2006).

LTP and LTD represent Hebbian synaptic plasticity mechanisms that function at single synapses to store particular information. Alternatively, homeostatic plasticity describes neurons' capacity to maintain network stability by globally adjusting synaptic strength upward or downward to prevent either complete silence or excessive activity (Turrigiano et al., 1998; Turrigiano, 2012; Pozo and Goda, 2010). Like Hebbian synaptic plasticity, homeostatic scaling regulates synaptic strength by adjusting AMPAR surface expression. During scaling up, synaptic levels of GluA1-containing AMPARs rise, including both GluA1 homomers and GluA1/GluA2 heteromers (Soares et al., 2013; Goel et al., 2011; Diering et al., 2014). Prolonged neuronal activity suppression (via TTX) multiplicatively increases synaptic AMPAR levels, with GluA1 exhibiting greater upregulation (scaling factor: 1.30) than GluA2 (1.16), indicating subunit-specific regulation in homeostatic plasticity (Venkatesan et al., 2020). During homeostatic scaling down, AMPARs containing GluA1 subunits are selectively internalized from synapses (Widagdo et al., 2017).

Synapses are highly dynamic structures, and AMPARs exhibit continuous movement even in the absence of neuronal activity (Nair et al., 2013; Archibald et al., 1998; Fukata et al., 2013). These receptors undergo constitutive trafficking between endosomes and the cell surface, with a half-life of 1–2 days (Passafaro et al., 2001; Ojima et al., 2021; O'Brien et al., 1998). The surface delivery of AMPARs is subunit-specific (Tian et al., 2015; Shi et al., 2001) and regulated by accessory proteins, such as transmembrane AMPAR regulatory proteins (TARPs).

Moving forward, the review will be focused specifically on AMPARs due to their sheer abundance and importance in excitatory synaptic transmission.

## AMPAR

AMPA Glutamate receptors are the primary excitatory neurotransmitter in the brain, mediating rapid signaling throughout the brain. AMPAR is composed of four subunits (GluA1-GluA4) and assembles to form tetrameric ion channels with two-fold symmetry. Each of these subunits contributes differently to receptor trafficking, selectivity, and kinetics (Miguez-Cabello et al., 2025). The pore-forming subunits (GluA1-GluA4) of AMPAR are made up of four distinct domains, the extracellular N-terminal domain (NTD) which functions for subunit assembly, synaptic localization, and receptor clustering (Rossmann et al., 2011; Kamalova and Nakagawa, 2021), the Ligand Binding Domain (LBD) which functions to bind L-glu and channel gating followed by the Transmembrane Domain (TMD), which is composed of three helix (M1, M3, and M4) and one re-entrance helix-loop (M2), this TMDs function for ion conductance by the AMPAR (Kamalova and Nakagawa, 2021; Hollmann et al., 1994), and finally the fourth domain is a cytoplasmic C-terminal domain (CTD) which is involved in controlling receptor anchoring, intracellular signaling, and trafficking (Kamalova and Nakagawa, 2021; Kim and Sheng, 2004). Based on the permeability of AMPAR for Calcium (Ca^2+^), they are functionally grouped into two types: Ca^2+^ permeable or Ca^2+^ impermeable AMPAR. The permeability of AMPAR for calcium is determined by the presence or absence of Q/R edited GluA2 subunit. GluA2-containing AMPARs that are Q/R site–edited predominantly in the central nervous system and are impermeable to Ca^2+^ (Miguez-Cabello et al., 2025; Seeburg and Hartner, 2003). Dysregulation of Q/R site editing in GluA2R has also been linked to the development of AD, both in human and mouse models (Wright et al., 2023; Khermesh et al., 2016). The number of AMPARs at any given synapse is subject to regulation by neuronal activity. The retention of AMPAR at the synaptic site involves three distinct steps: 1. exocytosis of the intracellular AMPARs, 2. Lateral diffusion of these receptors toward synaptic sites and 3. Anchoring at the synaptic site through interaction with scaffolding proteins (Opazo and Choquet, 2011).

## Auxiliary subunits associated with AMPARs

**Figure 5:**
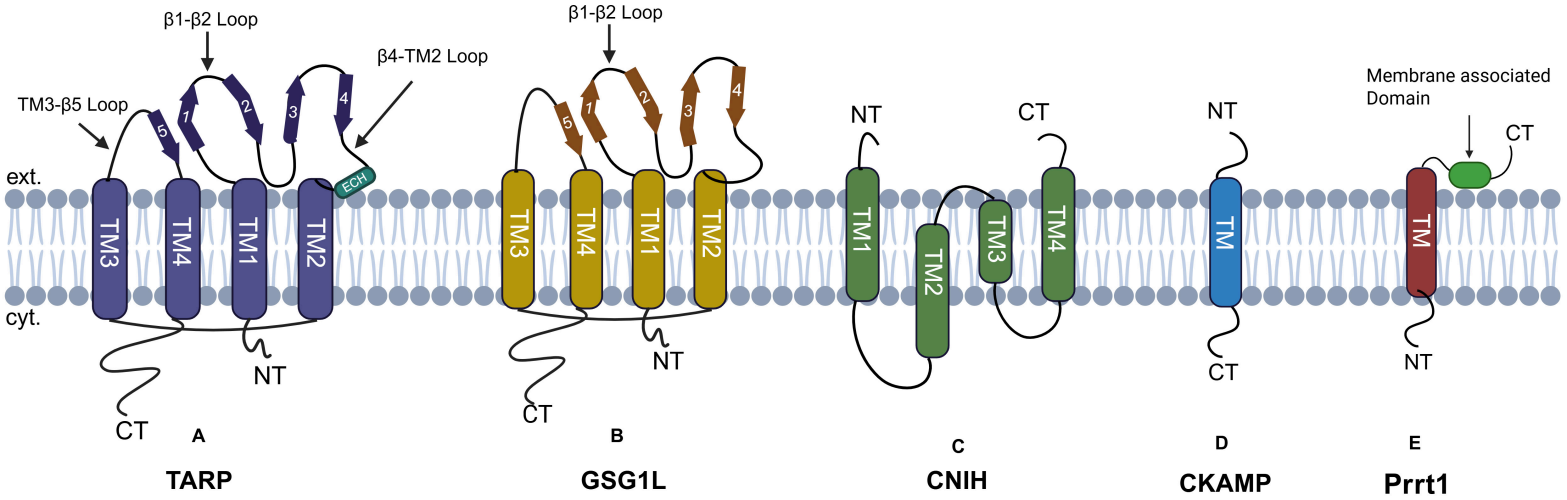
AMPARs auxiliary subunits: **(A)** TARP has four transmembrane domains, with both N and C-terminal domains facing the cytoplasm. The extracellular domain is composed of β-strands, an extracellular helix, and four flexible loops. **(B)** GSG1L has four transmembrane domains labeled as TM (1–4). Both the N and C termini of GSG1L face the cytoplasm, with the beta-sheet facing the extracellular space. **(C)** CNIH's protein has four transmembrane helices, with both N and C-terminal facing the extracellular space. **(D)** CKAMP has a single transmembrane domain, the N-terminal faces the extracellular space, and the C-terminal faces the cytoplasm. **(E)** SynDIG4 has a single transmembrane domain and a membrane-associated domain facing the extracellular space.

Although the native AMPAR complex is composed of the assembly of four GluA subunits, the functional AMPAR has auxiliary subunits associated with it. Some of the well-studied auxiliary subunits include TARPs (Tomita et al., 2005; Chen et al., 2000), cornichons (CNIHs) (Schwenk et al., 2009), CKAMP44 (Shisa9) (von Engelhardt et al., 2010), Shisa6 (Klaassen et al., 2016), SynDIG1/Prrt1, SynDIG4 (Kalashnikova et al., 2010), and GSG1L (Perozzo et al., 2023) as shown in Figure 5. These auxiliary subunits are known to add functional diversity for channel gating kinetics and receptor localization.

## TARPs

The two most abundant AMPAR auxiliary subunits in the hippocampus, cortex, and striatum are TARPs and CNIHs. Structurally, TARP proteins are claudin-like proteins that were identified as having protein homology to the γ-subunits of voltage-dependent calcium channels (VDCC), which are known to regulate the function of AMPARs in the post-synaptic membrane (Twomey et al., 2019a). Based on the sequence and function, TARPs are classified into three types: Type 1a (γ2, γ3), Type 1b (γ4, γ8), all of which are positive modulators of AMPAR, while Type 2 (γ5, γ7) exhibit distinct and diverse functions. *In situ* hybridization and western blotting experiments by Tomita et al. (2003) have shown that TARPs show a regional distribution in the brains, the highest level of TARP γ2 occurred in the cerebellum, γ-3 in the cerebral cortex, γ-4 in the olfactory bulb, and γ-8 in the hippocampus (Tomita et al., 2003). The primary point of interaction between AMPAR–TARP is TMD, where auxiliary subunits act as a scaffold around the AMPAR (Shaffer et al., 1987). TARPs subunits are known to regulate various properties of AMPAR, such as mean channel conductance, channel kinetics (Shaffer et al., 1987; Priel et al., 2005), polyamine sensitivity (Soto et al., 2007), opening probability (Priel et al., 2005), localization, and trafficking of AMPAR to PSD95 (Coombs and Cull-Candy, 2009). Experimental evidence from Opazo et al. (2010) has shown that phosphorylation of the C-terminal tail of Stargazin by CAMKII causes the diffusional trapping and accumulation of AMPARs at the synaptic site, enhancing the surface localization of AMPAR (Opazo et al., 2010). Mechanistically, they found that phosphorylation elongates the length of the C-terminal tail, facilitating binding even to the farthest-located AMPAR and PSD-95 contributing to the increased synaptic transmission (Hafner et al., 2015). Yamazaki et al. (2004) also found that the co-expression of GluA2 with stargazing enhanced the export of GluA2 from the Endoplasmic Reticulum (ER) and increased the surface expression of GluA receptors.

The assembly of AMPARs begins in the endoplasmic reticulum (ER), where GluA subunits first form homo or hetero dimers to form tetramers. Experimental evidence from Bedoukian et al. (2006) has given compelling evidence that TARPs associate with AMPARs in the ER at this early stage (Bedoukian et al., 2006). AMPARs undergo various post-translational modifications assisted by TARPs and interact with additional trafficking-associated proteins such as nPIST (Cuadra et al., 2004), MAP1 LC2 (Ives et al., 2004), and AP-4 (Matsuda et al., 2008), which are believed to assist in directing the receptor complex to the cell surface via vesicular transport. Co-immunoprecipitation experiments by Tomita et al. (2003) showed that the AMPARs co-immunoprecipitated with stargazing, suggesting that Stargazin strongly interacts with AMPAR. The PDZ-binding domains of type I TARPs, type II lacks it, located toward COOH of the terminus, interact with PSD-95 and immobilizes TARPs to the post-synaptic membrane, causing AMPARs to accumulate at post-synaptic sites (Coombs and Cull-Candy, 2009), contributing to the synaptic plasticity (Heine et al., 2008) and formation of macromolecular condensate, liquid-phase separation (Zeng et al., 2019). The PDZ-binding domain of TARP has a consensus site which acts as a substrate for various kinases, and phosphorylation of this site is known to disrupt the interaction with PSD-95, preventing synaptic clustering of AMPARs (Choi et al., 2002; Chetkovich et al., 2002).

Stargazin was first discovered in 1990 in A/J inbred mice line at the Jackson Laboratory. Mice homozygous for the autosomal recessive mutant form of the *Cacng2* gene in chromosome 15 displayed a distinct clinical behavior of frequently looking upward, so the researchers named the mutation “stargazer” (Noebels et al., 1990). The ectodomain of Stargazin is known to influence the gating of AMPAR, thus shaping the post-synaptic current. To quantify the glutamate-evoked current in the presence of Stargazin, Tomita et al. (2005) injected varying amounts of Stargazin and GluA1 in oocytes and measured glutamate-evoked currents. They found that GluA1, which shows no detectable current on its own, produces nearly maximal current when co-expressed with stargazin. Furthermore, they investigated whether stargazin influences both receptor trafficking and channel properties. To quantify the surface expression of GluA1, they tagged the hemagglutinin (HA) epitope into the extracellular region of GluA1. They observed that when oocytes were injected with 2, 1, or 0.1 ng of GluA1 cRNA, both the amount of GluA1 on the cell surface and the currents triggered by L-glu were reduced, while co-injection of stargazin cRNA with 0.1 ng GluA1 significantly enhanced glutamate-evoked currents. Notably, the increase in current was substantially greater than the increase in GluA1 surface expression, suggesting that stargazin enhances GluA1 function through a mechanism that is not solely dependent on receptor trafficking (Tomita et al., 2005).

Structurally, TARPs are composed of four transmembrane domains (TM1-TM4), with an intracellular N-terminal domain (NTD) preceding TM1. The extracellular domain is composed of β-strands, an extracellular helix, four flexible loops, and an intracellular C-terminal Domain (CTD), as shown in Figure 5. The TM3 and TM4 domain of TARPs directly interacts with M1 and M4 of adjacent AMPAR subunits (Twomey et al., 2016). As described earlier, AMPAR has a twofold symmetry which gives rise to two distinct TARP binding sites referred to as A'/C' and B'/D' sites (Zhao et al., 2016). This site has no relation to the A/C and B/D subunit positions defined within the AMPAR tetramer. The A'/C' binding sites are located beneath the ABD dimers and are more sterically constrained compared to the B'/D' binding sites, which are located below the ABD dimers. As a result, the specific binding position of TARPs within the AMPAR complex may influence how they modulate receptor function (Hansen et al., 2021).

TARPs act as a positive modulator of synaptic transmission; they act by slowing the desensitization and deactivation kinetics of AMPARs. The residues in the lower lobe of the AMPAR ABD are found to be critical for γ-2 mediated gating modulation and are hypothesized to interact with the extracellular domain of TARPs, slowing desensitization kinetics of GluA2 AMPARs (Twomey et al., 2019b). In type I TARPs, the residues within the β1–β2 loop of the extracellular domain are also responsible for slowing receptor desensitization (Hawken et al., 2017).

## Germ cell-specific gene 1-like (GSG1L) protein

GSG1L is another widely studied auxiliary subunit known to interact with GluA1 and GluA2 subunits of AMPAR (Keifer et al., 2017). GSG1L shows some structural similarity to TARPs, as shown in Figure 5. GSG1L expresses during later phase of development and is expressed in region specific manner in brain, GSG1L can either co-assemble with TARPs, Cornichons or as the sole auxiliary subunits. GSG1L slows the recovery of AMPARs from desensitized state through evolutionarily-conserved allosteric site unique to GSG1L (Perozzo et al., 2023). Schwenk et al. (2012) also found that this protein is associated with GluA2- or GluA4-containing AMPARs in dendritic spines of hippocampal pyramidal neurons, giving a clue that these proteins may possibly have a role in synaptic transmission. Shanks et al. (2012) found that GSG1L negatively modulates synaptic transmission; it works by stabilizing the desensitized state (Schwenk et al., 2012). Studies have shown that the two GSG1L subunits bind to the M1 and M4 of adjacent AMPARs, preferentially to the B0/D0 site (Twomey et al., 2019a, 2017).

## Cornichon (CNIHs)

Proteomic analyses have identified four cornichon family proteins (CNIH1-4 4); however, only CNIH2 and CNIH3 have been shown to associate with AMPAR (AMPAR) subunits (Schwenk et al., 2009). Herring et al. (2013) showed that CNIH-2/-3 selectively bind to GluA1 in hippocampal neurons, enhancing surface expression of GluA1A2 receptors (Herring et al., 2013). Cornichon has four transmembrane domains with both N and C termini facing the extracellular space, as shown in Figure 5. CNIH2 is expressed abundantly in the Hippocampus, striatum, and cortex (Schwenk et al., 2014). Previous studies in Drosophila, chickens, and cultured cells have characterized cornichon and its homologs as cargo exporters in the endoplasmic reticulum (ER) for members of the transforming growth factor α (TGFα) family (Castro et al., 2007; Bökel et al., 2006). Based on these findings, Shi and colleagues examined the potential role of CNIH2 in AMPAR trafficking. Their study demonstrated that CNIH2 facilitates the export of AMPARs to the cell surface (Shi et al., 2010). Their findings were further supported by Harmel et al. (2012) who found that the overexpression of CNIH-2 in HeLa and primary neurons increased the functional surface population of AMPARs. Investigation of the underlying mechanism unrevealed that the CNIH-2 subunits continuously shuttle between the endoplasmic reticulum (ER) and the Golgi apparatus. During this cycle, they pick up cargo proteins in the ER and facilitate their selective export through a coat protein complex II (COPII)-dependent mechanism (Harmel et al., 2012). CNIH3 and TARPs both bind to the surface formed by the M1 and M4 helices of neighboring AMPAR subunits, resulting in competition between them for the same binding site; this reduces the number of CNIH that can potentially bind with AMPARs (Herring et al., 2013). Like TARPs, CNIH proteins also directly modulate AMPAR function by enhancing L-glu sensitivity and prolonging both deactivation and desensitization (Schwenk et al., 2009; Coombs et al., 2012).

## Cystine-knot AMPA receptor-modulating proteins (CKAMPs)

CKAMPs constitute a family of four proteins, namely CKAMP39 (shisa8), CKAMP44 (shisa9), CKAMP52 (shisa6), and CKAMP59 (shisa7). Structurally, they are classified as type I transmembrane proteins, with the cysteine-rich N-terminal domain oriented toward the extracellular space and the C-terminal domain, with a PDZ type II binding motif, facing the cytoplasm. The extracellular cysteine-rich domain and short stretch of 20 amino acids immediately downstream of the transmembrane domain is known to interact and modulate AMPARs (Khodosevich et al., 2014), while the PDZ type II binding motif act as anchoring site proteins for such as PSD95, GRIP1, MPP5, PICK1 and Lin7b (Klaassen et al., 2016; Khodosevich et al., 2014; Kunde et al., 2017). CKAMPs family members show regional expression profiles within the brain, CKAMP39 is expressed in granule cells of the cerebellum and olfactory bulb, CKAMP44 is abundantly expressed in the dentate gyrus and the glomerular layer of the olfactory bulb, CKAMP52 is expressed in the principal cell layers of all hippocampus, and the Purkinje layer of the cerebellum, KAMP44 and CKAMP52 only colocalize with glutamatergic synapses. While CKAMP59 mRNA is expressed in the cortex, striatum, and principal cell layers of the hippocampus, the granule cell layer of the olfactory bulb (von Engelhardt, 2019). CKAMP44 enhances the sensitivity of AMPARs to L-glu, slows the rate of deactivation, accelerates desensitization, and delays recovery from the desensitized state. These effects may be due to its ability to stabilize the closed-cleft conformation of the agonist-binding domain (ABD), favoring the desensitized state (von Engelhardt et al., 2010).

## Synapse differentiation-induced gene 4 (SynDIG4)/Prrt1

SynDIG4/Prrt1 is a type II transmembrane protein that is known to colocalize with AMPARs GluA1 subunit at synapses and extrasynaptic sites, modulating the AMPARs' activity. Prrt1 was initially identified as a key gene involved in synapse differentiation (Kalashnikova et al., 2010; Kirk et al., 2016). Prrt1 has an N-terminal domain facing the cytoplasm, a transmembrane domain, and a membrane-associated C-terminal domain as shown in Figure 5. Prrt1 is known to interact with all of the AMPAR subunits GluA1-GluA4. This interaction is mediated by the transmembrane domain and intracellular loop (Martin et al., 2021). SynDIG4 is known to act by slowing the deactivation and desensitization kinetics of AMPARs in a subunit-dependent manner. SynDIG4 Knockout mice, therefore, have shown reduced AMPAR-mediated mEPSCs (Matt et al., 2018).

### Disease associated with AMPAR complexes

Auxiliary subunits of AMPAR are known to interact directly or indirectly with AMPAR subunits and several synaptic molecules that modulate receptor trafficking, localization, and gating kinetics; their dysregulation has been directly linked to the pathology of various neurological disorders.

## Amyotrophic lateral sclerosis (ALS)

Amyotrophic lateral sclerosis (ALS) is a progressive neurodegenerative disorder that leads to the degeneration of motor neurons projecting from the motor cortex, brainstem, and the spinal cord. Several studies have suggested that AMPARs play a major role in slow and selective neurodegeneration of motor neurons as seen in ALS. To date, riluzole—a glutamate-modulating drug with anti-excitotoxic effects—remains the only therapy shown to slow ALS progression and prolong survival by roughly 2–3 months (Brooks, 2009; Bensimon et al., 1994), reinforcing the notion that glutamate-driven excitotoxicity is central to ALS pathogenesis. Although NMDAR-mediated mechanisms have traditionally been implicated in glutamate-induced excitotoxicity, emerging evidence suggests that in ALS, calcium-permeable AMPARs play a predominant role in mediating neuronal excitotoxicity. The exact mechanism underlying neuronal excitotoxicity in ALS has not yet been fully understood, but it has been found that the overactivation of AMPARs causes mitochondrial Ca^2+^ overload, leading to mitochondrial damage and production of Reactive Oxygen Species (ROS) (Williams et al., 1997; Carriedo et al., 1998, 2000). Neurons show selective vulnerability to excessive Ca^2+^ influx; motor neurons have lower expression of Ca^2+^ binding proteins, limiting their Ca^2+^ buffering properties, which makes them more susceptible to AMPAR-mediated excitotoxicity (Van Den Bosch et al., 2006). Permeability to Calcium by AMPARs is determined by the presence of the GluA2 subunit; its presence makes AMPARs impermeable to Ca^2+^ (Keinänen et al., 1990). But as mentioned earlier in the review, GluA2-containing AMPARs have now been shown to have permeability for calcium ions (Miguez-Cabello et al., 2025). GluA2 subunit undergoes post-translational modification at the Q/R site of the M2 domain, where glutamine is substituted by arginine. It is the presence of this edited Arginine in the central position that renders calcium impermeability to the GluA2 bearing AMPARs (Seeburg and Hartner, 2003). In a study conducted by Kawahara et al., they reported a significant reduction in RNA editing at the Q/R site of the GluA2 subunits in spinal motor neurons of patients with sporadic ALS, suggesting that this alteration may contribute to the development and progression of the disease (Kawahara et al., 2004).

## Alzheimer's disease (AD)

Alzheimer's disease (AD) is a progressive neurodegenerative disorder that predominantly impairs memory and cognitive function. The pathological hallmarks of AD include the accumulation of extracellular amyloid-β (Aβ) plaques and the formation of intracellular neurofibrillary tangles composed of hyperphosphorylated tau protein (Hardy and Higgins, 1992; Glenner and Wong, 1984). During the early stages of AD, synaptic deterioration emerges as a key pathological feature, and AMPARs are the principal glutamate receptors that mediate fast excitatory neurotransmission. Thus, synaptic deterioration is strongly linked to the impaired function of α-amino-3-hydroxy-5-methyl-4-isoxazolepropionic acid (AMPA) receptors. Dysfunction of these receptors disrupts excitatory neurotransmission and synaptic plasticity, processes that are critical for learning and memory, thereby contributing to the early cognitive deficits observed in AD (Ning et al., 2024). As discussed earlier, AMPARs are composed of GluA1-GluA4 subunits, and Aβ has been known to bind to the C-terminal tail of unedited GluA2 subunits, which leads to a reduction in the expression of surface AMPARs, weakening the strength between synapses (Babaei, 2021). The exact molecular mechanism that leads to the decrease in the expression of AMPARs has not yet been fully elucidated, but a study conducted by Zhang et al. (2018) reported that the presence of Aβ both in culture and brain of AD patients showed enhanced ubiquitination of surface AMPARs, facilitating receptor internalization and degradation causing LTD. Enhanced ubiquitination is mediated by upregulation of Nedd4, an E3 ligase, and downregulation of deubiquitinase USP46 (Zhang et al., 2018). Aβ not only alters the ubiquitination of AMPARs but is also known to alter the acetylation of AMPARs in AD conditions. Normally, AMPARs are subjected to lysine acetylation by p300 acetyltransferase, conferring them higher stability, reduced receptor internalization and degradation (Wang G. et al., 2017). Under AD conditions, AMPARs are hypoacetylated, leading to a reduction in surface AMPARs and contributing to synaptic weakening and impaired cognition, as seen in AD (O'Connor et al., 2020). In addition to ubiquitination, Aβ may also impact the surface diffusion of AMPARs. Accumulation of amyloid-β (Aβ) within neurons has been known to mislocalize and internalize the L-glu transporter in astrocytes, decreasing L-glu clearance (Matos et al., 2008; Li et al., 2009; Lanznaster et al., 2017). Aβ also increases L-glu spillover through α7 nicotinic acetylcholine receptors (a7nAChR) (Zhang et al., 2022; Kabogo et al., 2010; Hascup and Hascup, 2016). This excessive L-glu leads to aberrant activation of extrasynaptic GluN2B-containing NMDARs, subsequently activating downstream Rap-p38 MAP, protein phosphatases PP1, and calcineurin, inducing AMPAR endocytosis and excitotoxicity, contributing to progressive neuronal damage as seen in AD (Guntupalli et al., 2016). The modulation of AMPARs has emerged as a promising therapeutic strategy for addressing cognitive deficits associated with AD. Preclinical studies have demonstrated the efficacy of AMPAR-positive modulators in enhancing synaptic transmission and improving memory functions. One such compound, LY451395, has been shown to act as a positive allosteric modulator of AMPARs. In animal models, systemic administration of LY451395 increased AMPAR-mediated synaptic responses in the hippocampus and significantly improved performance in memory-based behavioral tasks. However, despite promising preclinical outcomes, clinical trials in patients with mild to moderate AD failed to show significant cognitive improvement following an 8-week administration of LY451395. Nevertheless, these studies underscore the potential of AMPAR modulation in AD therapy (Chappell et al., 2007; Chang et al., 2012).

## Epilepsy

Epilepsy ranks among the most prevalent neurological disorders worldwide. The characteristic features of Epilepsy include recurrent, unprovoked seizures resulting from brief, abnormal bursts of electrical activity in the brain that alter behavior, consciousness, cognition, and/or movement. These episodes reflect a temporary disruption of the brain's excitatory-inhibitory balance, detectable through EEG monitoring. (Milligan, 2021). Elevated levels of extracellular L-glu concentrations have been seen in epilepsy patients (Sarlo and Holton, 2021). Evidence shows that AMPAR antagonists effectively suppress seizures, indicating an important role of glutamatergic signaling in epilepsy pathogenesis (Chang et al., 2016; Celli and Fornai, 2021; Barker-Haliski and White, 2015). Genetic screening of epilepsy patients has identified Nedd4-2 mutations, an epilepsy-associated gene encoding a ubiquitin E3 ligase that regulates neuronal activity via GluA1 ubiquitination. All three identified missense mutations disrupt GluA1 ubiquitination, failing to reduce surface GluA1 levels and also spontaneous neuronal activity, explaining the heightened electrical activity in the brain (Zhu et al., 2017). Studies also showed a decreased number of AMPARs in epileptic patients compared to normal people (Eiro et al., 2023). Also, excessive glutamate receptor activation elevates intracellular calcium levels, contributing significantly to neuronal death in epilepsy. After a neurologically adverse incident like an epileptic seizure, GluA2 AMPAR expression drops, promoting the formation of calcium-permeable, GluA2-lacking AMPARs that may amplify glutamate-mediated neurotoxicity (Lorgen et al., 2017).

## Limbic encephalitis and Rasmussen's encephalitis

Limbic encephalitis involves autoimmune-mediated inflammation targeting the limbic system and other brain regions. Some limbic encephalitis patients harbor autoantibodies against GluA1, GluA2, or GluA1/GluA2 subunits. These autoantibodies target the N-terminal (NTD) and ligand-binding (ABD) domains of GluA1/2 without binding specific epitopes (Gleichman et al., 2014). Anti-GluA1/2 autoantibodies promote synaptic AMPAR internalization, causing chronic AMPAR dysfunction and impaired synaptic plasticity (Peng et al., 2015; Lai et al., 2009; Haselmann et al., 2018). Disrupted synaptic plasticity underlies the memory deficits observed in mouse models infused with patient-derived anti-GluA2 autoantibodies (Haselmann et al., 2018). While these antibodies primarily target GluA1 and GluA2 epitopes, they also reduce synaptic levels of GluA3-containing AMPARs (e.g., GluA2/3 heteromers) (Peng et al., 2015; Lai et al., 2009).

Anti-GluA3 autoantibodies were initially identified in a Rasmussen's encephalitis patient (Rogers et al., 1994), a severe pediatric condition characterized by seizures, hemiparesis, motor deficits, and cognitive decline (Bien et al., 2005). These antibodies have since been associated with additional seizure disorders (Levite, 2014). Multiple anti-GluA3 autoantibodies, including those from Rasmussen's encephalitis, bind to the N-terminal domain (NTD) residues 372–395 of GluA3 (Mantegazza et al., 2002; Levite et al., 1999; Ganor et al., 2005a). These antibodies function as positive allosteric modulators, exerting pathogenic effects through both direct agonist activity and complement-dependent cytotoxicity (Levite et al., 1999; Whitney and McNamara, 2000; Twyman et al., 1995; He et al., 1998; Cohen-Kashi Malina et al., 2006; Carlson et al., 1997). Animal models immunized with peptides containing the anti-GluA3 epitope develop behavioral impairments and neuronal degeneration (Goldberg-Stern et al., 2014; Ganor et al., 2005b, 2014). Current standard treatments for Rasmussen's encephalitis remain immunosuppressive therapy and surgical hemispherectomy of the affected cerebral hemisphere.

## Autism spectrum disorder (ASD)

Autism spectrum disorder (ASD) is a complex neurodevelopmental disorder affecting people's ability to communicate, interact, and learn. Genes implicated in ASD are often involved in fundamental cellular pathways, including protein synthesis, cell proliferation, and synaptic function, highlighting the molecular complexity underlying the pathophysiology of ASD (Ozonoff et al., 2005; Lin et al., 2016; Hussman et al., 2011). ASD has become a widespread and socially significant neurodevelopmental condition, currently affecting approximately 1.5% of the population (Lyall et al., 2017; Kanner, 1968).

The causes of ASD consist of a wide range of contributing factors, such as *de-novo* mutations and microdeletions of GRIA2 gene (Rylaarsdam and Guemez-Gamboa, 2019; Salpietro et al., 2019); environmental and acquired influences during the pre-, peri-, and postnatal periods (Wang C. et al., 2017); and the polygenic co-inheritance of, asymptomatic haplotypes or polymorphisms that may converge to disrupt neural circuit homeostasis (Rubenstein and Merzenich, 2003). Among these are the structural components critical to synaptic integrity and brain circuit function. Particularly those involving AMPAR-mediated neurotransmission. Some pathways demonstrate selective enrichment in cerebro-cerebellar regions, hinting at their role in ASD (Ebrahimi-Fakhari and Sahin, 2015; Bagni and Zukin, 2019). Findings have shown a general upregulation of AMPAR mRNA transcripts, coupled with a selective reduction in AMPAR protein levels within the cerebellum in individuals affected by autism (Purcell et al., 2001). Studies have also identified a potential molecular pathway underlying this disruption: loss of function in UBE3A, a gene implicated in both autism and Angelman syndrome, was shown to increase AMPAR internalization, thereby attenuating synaptic AMPAR availability and reducing LTP (Greer et al., 2010). Genetic mutations in *P-Rex1*, a gene involved in regulating AMPAR endocytosis, have been identified in individuals with autism, and preclinical models lacking this gene exhibit behavioral phenotypes consistent with core features of ASD, including social deficits and reduced behavioral flexibility (Li et al., 2015). *Neurobeachin*, a gene identified in autism-associated cohorts, has revealed pronounced disruptions in dendritic spine morphology accompanied by a reduction in miniature excitatory post-synaptic currents (mEPSCs) (Niesmann et al., 2011). In contrast, studies of *Rbm8a*, a gene involved in non-sense-mediated mRNA decay and similarly linked to autism, have demonstrated abnormally elevated mEPSC frequency (Alachkar et al., 2013). Genetic mouse models with targeted deletions of synaptic scaffolding proteins such as Shank3 exhibit a significant reduction in AMPAR-mediated current, contributing to the synaptic deficit. This finding supports the hypothesis that altered AMPAR signaling contributes to the neurodevelopmental and behavioral phenotypes observed in ASD (Lee et al., 2017; Jaramillo et al., 2020). These findings hint toward a role of AMPARs in ASD pathogenesis due to their crucial role in synaptic transmission and generation of EPSCs.

## Perspectives

Glutamatergic receptors, particularly AMPARs, constitute one of the most critical molecular families in the central nervous system. Their biophysical properties, subunit composition, interaction partners, and membrane dynamics exert a direct influence on synaptic function, plasticity, and the pathophysiology of numerous brain-related disorders. Changes in AMPAR expression, trafficking, and clustering are increasingly recognized as molecular hallmarks of neurodevelopmental, neuropsychiatric, and neurodegenerative diseases. Over the past three decades, a substantial body of knowledge has accumulated regarding the molecular composition and biochemical behavior of AMPARs. These studies have identified a range of endogenous ligands, mapped their expression profiles across brain regions, and explored their interaction networks through genomic, proteomic, and biochemical paradigms. Classical structural and biophysical approaches, including crystallography, electrophysiology, and fluorescence-based imaging, have offered deep insight into AMPAR function at synaptic membranes. However, recent studies are revealing a new layer of complexity, with a growing emphasis on the spatial distribution of AMPARs within sub-synaptic and extrasynaptic domains, their dynamic trafficking kinetics, and the emergent principles of allosteric gating.

One emerging theme is the spatial regulation of AMPAR clustering at nanodomains, which modulates both the amplitude and frequency of excitatory post-synaptic currents. Subsynaptic organization is now known to be governed by multiple mechanisms, including activity-dependent anchoring, scaffolding interactions, and homeostatic scaling. These processes are not merely passive; they exert non-linear effects on receptor gating, ligand binding, and signal integration. For instance, changes in receptor density and localization in nanodomains influence ligand binding kinetics, desensitization rates, and synaptic current summation, collectively reshaping synaptic strength and plasticity. Understanding this spatial and kinetic heterogeneity is increasingly important for future therapeutic targeting. The complexity of AMPAR function cannot be captured by traditional pharmacology alone. Future strategies will likely require multi-pronged approaches that integrate molecular modeling, AI-guided drug discovery, and high-throughput screening platforms. These methods can be informed by detailed structural insights from cryo-electron microscopy and super-resolution imaging and validated using patch-clamp electrophysiology and optogenetic assays in physiologically relevant models.

The mounting evidence points to AMPARs as early molecular transducers of activity-dependent synaptic changes. As such, they represent promising targets for therapeutic modulation in early-stage cognitive dysfunction and neurodegenerative disease. One forward-looking direction involves engineering synthetic ligands that can selectively modulate AMPAR activity in a brain region-specific manner, effectively designing chemical switches to silence or activate particular excitatory circuits. Additionally, advances in genome editing technologies such as CRISPR-Cas9 have opened new possibilities to modify AMPAR genes in the adult brain with cell-type and region-specific precision. This raises the exciting potential for molecular engineering of customized receptor variants that could restore or tune synaptic function in disease contexts. By combining synthetic biology, structural pharmacology, and next-generation imaging and editing tools, the next decade may offer transformative strategies for regulating AMPAR activity with unprecedented precision and efficacy in the context of brain disorders.
